# How an Elevated Creatinine Level Can Deter the Diagnosis of Acute Aortic Dissection

**DOI:** 10.7759/cureus.2057

**Published:** 2018-01-12

**Authors:** Mohamed A Mohamed, Kewan A Hamid

**Affiliations:** 1 Michigan State University College of Human Medicine; 2 Department of Combined Internal Medicine-Pediatrics, Hurley Medical Center, Michigan State University College of Human Medicine

**Keywords:** acute aortic dissection, cardiovascular emergencies, emergency imaging, hematuria, hypertensive emergency

## Abstract

Acute aortic dissection (AAD) classically manifests with sudden, severe chest pain radiating to the back or abdomen, often described as ripping or tearing sensation. Considering its abrupt onset, the diagnosis requires a high index of suspicion prompting immediate imaging using computed tomography (CT) with contrast. However, the use of contrast is a relative contraindication in the patients with renal compromise and acute care physicians are often deterred from contrast use in these patients. Herein, we present an unusual case of hematuria as the presenting symptom of a developing the Stanford type-A AAD.

A 65-year-old female presented with sudden, severe chest pain radiating to her lower back. She reported that her urine color was 'pink' on the previous day and was becoming more 'red-colored' as the day progressed. The next morning, she began feeling a 10/10 crushing-type chest pain that was relieved when she lay on her left side and was associated with nausea, vomiting, and diaphoresis. The urine analysis revealed gross hematuria. The laboratory findings revealed a creatinine of 1.3. Due to her elevated creatine levels and possible acute kidney injury, a computed tomography (CT) without contrast was performed initially, which did not reveal an AAD. However, the index of suspicion was still high for the AAD, after prompt discussions about the risk of using contrast and contrast nephropathy versus the risks of potential complications, if AAD was missed. Further evaluation with CT of the chest and abdomen, with contrast, was obtained with the patients' consent, which revealed a Stanford type-A AAD starting proximally from the aortic arch and extending to the common iliac.

In conclusion, the clinical presentations of AAD are more heterogeneous. Hematuria in the presence of high index of suspicion and symptoms of AAD could indicate the extension of the involvement of the renal arteries. Prompt CT with contrast may be indicated despite relative contraindications from the laboratory findings.

## Introduction

Acute aortic dissection (AAD) is not a frequently encountered condition, with an incidence of up to 3.5 per 100,000 person-years [1]. The classical clinical manifestations of AAD are sudden, severe chest pain radiating to the back or abdomen and often described as ripping or tearing sensation. However, a 17-year report from the International Registry of Aortic Dissections (IRAD) suggests that the presentations of AAD are heterogeneous, with reports that up to 17% of the patients are asymptomatic [2]. Due to its abrupt onset and diagnostic difficulty, a high index of the clinical suspicion is vital to improve the survival in AAD, especially in the cases with atypical presentations. Herein, we present an unusual case of hematuria as the first symptom of a developing Stanford type-A AAD.

## Case presentation

A 65-year-old female presented with severe chest pain radiating to the back. The day prior, she noticed that her urine was 'pink' and was becoming more 'red-colored' throughout the day. That morning, she began feeling a 10/10 crushing-type chest pain, which improved only when she lay on her left side. Her symptoms were also associated with nausea, vomiting, and diaphoresis. Pertinent past medical history included uncontrolled hypertension, and noncompliance with the medications, back pain and a 55-year history of tobacco abuse. She reported that she did not take her antihypertensive medications for two months due to the lack of follow-up with her primary care physician. She denied the history of cocaine or any other illicit substance.

On presentation, her vitals were as follows: blood pressure (BP) from the right arm 262/76 mmHg; heart rate 57 beats/min; respiratory rate 20 breaths/min; and oxygen saturation (SaO2) 100% on room air. The initial chest radiograph was unremarkable and did not show a widened mediastinum. The urine analysis revealed gross hematuria. The laboratory findings included a blood urea nitrogen (BUN) of 19, creatinine of 1.3 with unknown baseline due to her urgent condition, which was unestablished at our center. She also had the mild elevation of liver enzymes. The initial troponin was 0.01, but increased to 0.17 after three hours. Her electrocardiogram (ECG) was suggestive of a non-ST elevation myocardial infarction (NSTEMI). Due to her elevated creatine levels, the computed tomography (CT) with contrast was not performed. The patients' blood pressure became controlled at 152/76 with the intravenous (IV) hydralazine and IV fluids, morphine, low-dose nitroglycerin, and aspirin was given. Heparin was not given due to elevating troponins.

**Figure 1:**
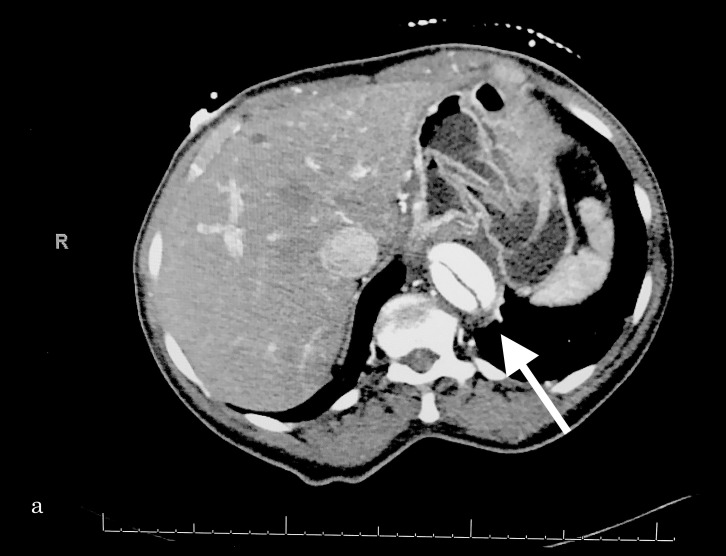
Transverse section demonstrating the aortic dissection near the liver (right).

**Figure 2:**
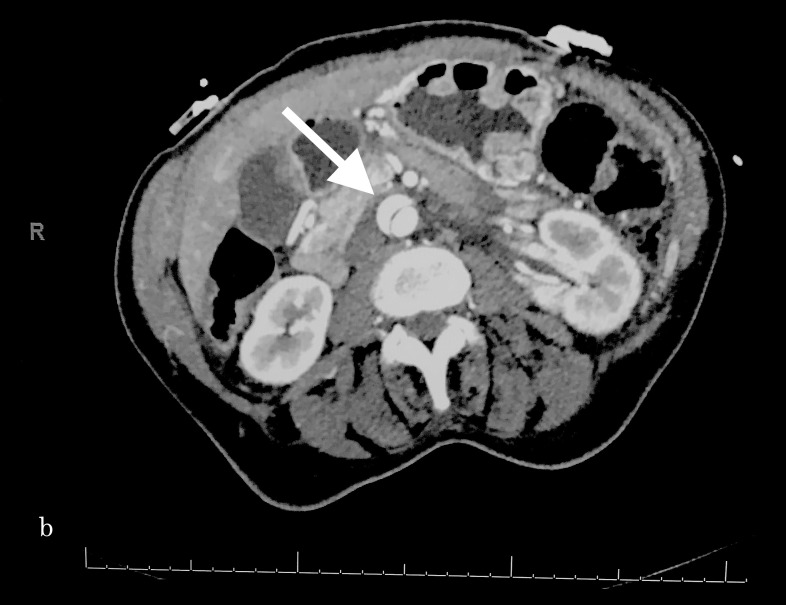
Transverse section demonstrating the aortic dissection at the level of the kidneys bilaterally.

**Figure 3:**
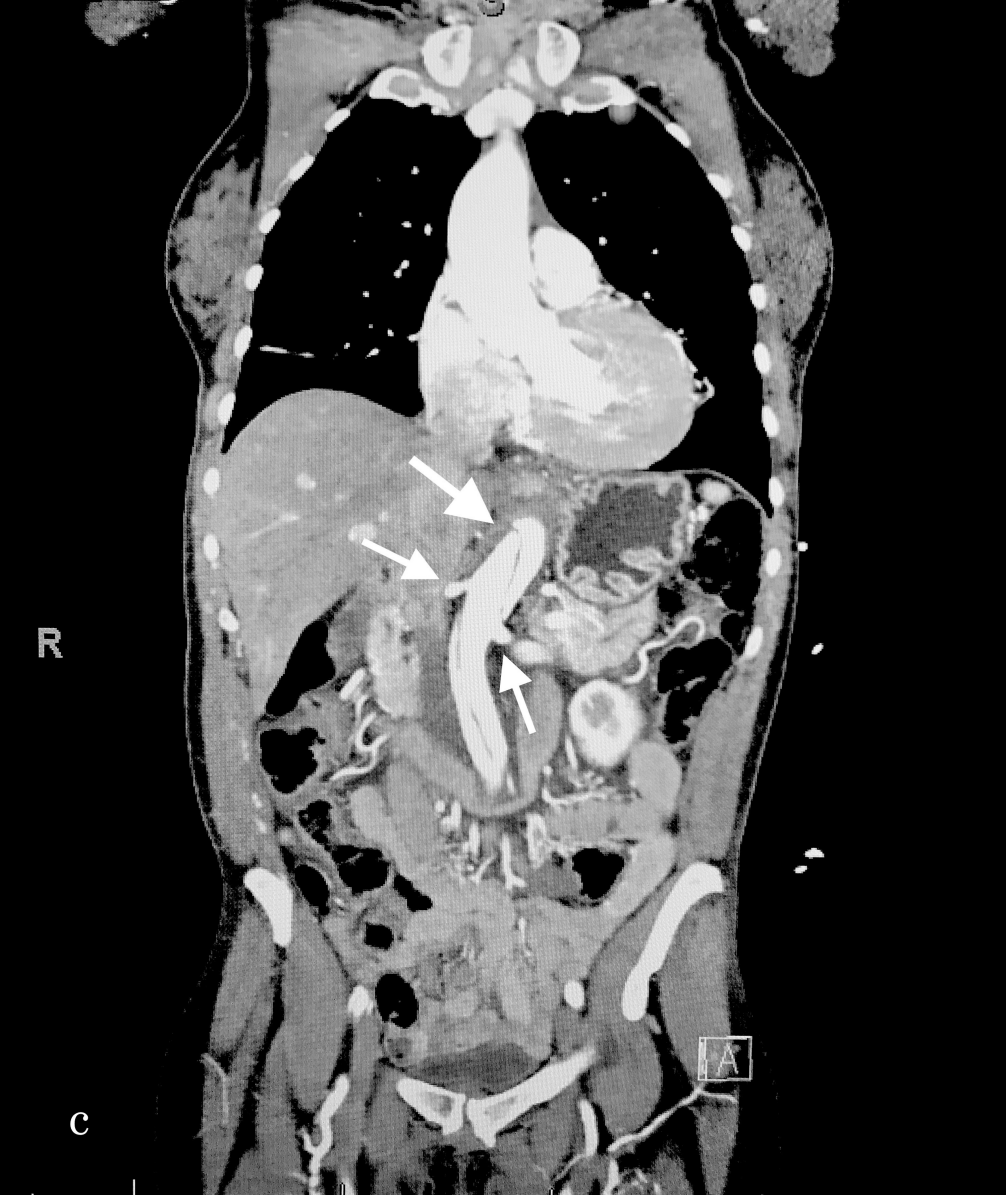
The frontal section displaying the dissection involving the renal arteries.

Due to increased suspicion of an AAD, prompt discussions about the risk of using contrast and the risks of not ruling out an AAD were initiated. The patient agreed to further evaluation, with the CT using contrast, which revealed a Stanford type-A AAD starting proximally from the aortic arch and extending to the common iliac (Figures 1-3). Subsequently, she was immediately transferred to a higher echelon of care for the surgical management. On follow up the next day, we were informed that the patient had expired due to her extensive organ failure that was unsalvageable.

## Discussion

In the past two decades, our knowledge of AAD has increased, which has subsequently improved the survival of these patients. In 2015, the IRAD examined 17-year trends, outcomes of 4,428 patients with AAD and found that there had been a decrease in the hospital mortality of AAD [2]. This observation is most likely due to rapid recognition and prompt intervention. Due to our clinical suspicion, the diagnosis was made and the patient was immediately transferred to the surgical management.

Renal ischemia, is a major complication of aortic dissection, with about 50% of the patients with renal infarction presenting with hematuria. However, hematuria is only present in 18% of the isolated renal artery dissections, with the most common presentations being the flank pain (77%) and hypertension (100%) [2]. Our patient did not have flank pain but did present with severe hypertension, consistent with the literature. Whether or not the hematuria was secondary to the AAD is unclear. In theory, it is reasonable to assume that the hematuria was a direct consequence of the dissection involving the renal arteries as seen in with our patient.

The mechanism of acute hematuria is unclear, though renal ischemia secondary to decreased blood flow has been theorized. Because dissection involving the renal arteries result in an abrupt onset of renal ischemia, the presence of hematuria and/or flank pain are of diagnostic and prognostic importance [3]. Our patient’s initial presentation was a one-day history of hematuria and then developed the chest pain radiating to the back, which suggests that either renal ischemia developed quickly or there was more severe involvement of the renal arteries compared to the rest of the dissection. From our review, we observed that the left renal artery was more frequently affected than the right. This is probably due to the right renal artery orifice being more anterior than the left. Thus, the right renal artery is only involved, if there is the substantial circumferential extension of the AAD [3-4]. From our review, the diagnosis of several patients was extremely delayed, often until hemodynamic stability is compromised (Table 1) [5-10].

**Table 1:** Summary of the cases of the acute aortic dissections presenting with hematuria. Yr: year, F/M: female/male, PMH: past medical history, BP: blood pressure, A/B: Stanford type, SCA: subclavian artery, BCA: brachiocephalic artery, AA: ascending aorta, DIC: disseminated intravascular coagulopathy, BUN: blood urea nitrogen, Cr: creatinine, AST/ALT: liver enzymes, CTD: cardiac tamponade.

Table 1. Summary of the cases of acute aortic dissections presenting with hematuria								
Author, yr	Age, sex	PMH	BP	A/B	Extent	Time, days	Symptoms, outcome and comments	
Our patient	65, F	HTN	262/76	A	Aortic arch - Common iliac	1	Hematuria, chest & back pain	
Cui et, al. 2015 [5]	63, M	HTN	n/a	B	L. SCA - Common iliac	< 1	Hematuria, diffuse petechiae; Developed DIC	
Kodama, et al. 2013 [8]	49, F	none	182/66	B	L. SCA - Common iliac	5	Hematuria, flank pain; Elevated CRP	
Ngan, et al. 2006 [10]	17, M	none	140/79	B	L. SCA - Common iliac	< 1	Hematuria, flank & abdominal pain, hematemesis, melena; Died; BUN 18, Cr 3.9, ALT 2136	
Jenq, et al. 2006 [7]	44, F	none	n/a	B	n/a	n/a	Hematuria, pyuria, flank pain; died; ischemic small bowel & ascending colon	
Tarif, et al. 2002 [6]	41, M	none	210/110	B	L. SCA - Common iliac	< 1	Hematuria, flank & back pain; elevated AST/ALT, Cr 2.4	
Demos, et al. 1981 [9]	58, M	none	160/90	A	L. BCA - Common iliac	2	Hematuria, flank, epigastric, & chest pain; died; pericardial dissection & CTD; BUN 28	
Demos, et al. 1981 [9]	53, M	n/a	130/74	A	AA - Renal arteries	7	Hematuria, flank & chest pain; BUN 23	
Demos, et al. 1981 [9]	55, M	none	132/80	A	L. SCA - Common iliac	10	Hematuria, flank, back & chest pain; died; diagnosed on autopsy	

The clinical presentations of the AAD are more variable than the classical findings, with up to 10% of the patients only present due to the secondary complications [8]. In our case, our patient had laboratory findings suggestive of the kidney insult, which deterred the clinician away from using contrast with imaging. Due to the rarity of AADs presenting with hematuria, the pain that is radiating to the back is often misdiagnosed as flank pain. This uncharacteristic set of circumstances makes this condition a diagnostic challenge. Furthermore, the mortality rate in this subset of the patients is high with reports of up to 50% (± 11%), most likely due to delayed diagnosis [4].

There are several risk factors for the AAD that have been identified. In the classical elderly patient with AAD, often a history of hypertension and atherosclerosis is present, which is similar to our patient. Though more common in older patients, the AAD occurring in younger patients are usually associated with some congenital cardiovascular disorders, such as coarctation of the aorta and aortic valvular stenosis, or connective tissue disorders [2, 10]. From our literature review, only two patients had hypertension as a possible risk factor, while the rest were previously healthy. Suggesting, that the true cause of the AADs may be multi-factorial.

## Conclusions

The clinical presentations of the AAD are variable and may be more heterogeneous than previously reported. Hematuria in the presence of the classical symptoms of AAD should trigger an index of suspicion for renal artery involvement. The prompt CT with contrast may be indicated despite relative contraindications from the laboratory findings, particularly in the patients with unknown baseline creatinine. The timely management is essential for the improved survival of these patients.
